# Postpartum Contraceptive Use and Its Determinants in Ethiopia: A Systematic Review and Meta-analysis

**DOI:** 10.1155/2020/5174656

**Published:** 2020-01-04

**Authors:** Tsegaye Mehare, Birhanie Mekuriaw, Zelalem Belayneh, Yewbmirt Sharew

**Affiliations:** ^1^Department of Biomedical Science, College of Medicine and Health Science, Dilla University, Dilla, Ethiopia; ^2^Department of Psychiatry, College of Medicine and Health Science, Dilla University, Dilla, Ethiopia; ^3^Department of Midwifery, College of Health Science, Debre Markos University, Debre Markos, Ethiopia

## Abstract

**Background:**

Postpartum contraceptive use is defined as the avoidance of short spaced pregnancies and unintended pregnancy through the first 12 months after delivery. In Ethiopia, different studies have been conducted to assess the prevalence of postpartum contraceptive use and associated factors. The findings of these studies were inconsistent and characterized by great variability. Therefore, the aim of this systematic review and meta-analysis was to estimate the pooled prevalence of postpartum contraceptive use and determinants in Ethiopia using the accessible studies.

**Methods:**

The articles were identified through electronic search of reputable databases (MEDLINE through PubMed, EMBASE, HINARI, Science Direct, and Cochrane Library) and the hand search of reference listed in previous prevalence studies to retrieve more. 18 articles are included based on a comprehensive list of inclusion and exclusion criteria. Two authors independently extracted all necessary data using a standardized data extraction format. STATA 14 statistical software was used to analyze the data. The Cochrane *Q* and *I*^2^ test were used to assess the heterogeneity between the studies. A random effects model was calculated to estimate the pooled prevalence of postpartum contraceptive use. Moreover, the determinants for family planning use were reviewed.

**Results:**

The pooled prevalence of family planning use among mothers during the postpartum period in Ethiopia was 48.11% (95% CI: 36.96, 59.27). Besides, subgroup analysis revealed that the highest family planning use prevalence among postpartum mothers was observed in Addis Ababa, 65.41 (95% CI: 48.71, 82.11). Resumed sexual activity: 7.91 (95% CI: 4.62, 13.55), antenatal care: 4.98 (95% CI: 2.34, 10.21), secondary school and above level of maternal education: 3.53 (95% CI: 1.67, 7.45), postnatal care: 3.16 (95% CI: 1.7, 5.88), menses resumption: 3.12 (95% CI: 1.52, 6.39), and ≥6 months of postpartum period: 2.78 (95% CI: 1.97, 3.93) have shown a positive association with the use of family planning among mothers in the postpartum epoch.

**Conclusions:**

In this study, family planning use among mothers of the postpartum period in Ethiopia was significantly low compared to the existing global commendation on postpartum contraceptive use. Resumed sexual activity, antenatal care, secondary and above level of maternal education, postnatal care, menses resumption, and postpartum period ≥ 6 months were found to be significantly associated with postpartum contraceptive use.

## 1. Introduction

Maternal health problems remain a major global concern since pregnancy and childbirth are the leading causes of morbidity and mortality among reproductive age women [1]. The World Health Organization (WHO) describes the postnatal period as the most critical and yet the most neglected phase in the lives of mothers and babies; most deaths occur during the postnatal period [2]. Worldwide, there are 265 million unwanted pregnancies, 110 million unnecessary abortions, 590,000 avoidable maternal deaths, and 8 million preventable infant deaths [3]. A study on global burden estimates 292,982 maternal deaths that occurred during 2013, and almost 99% of these deaths happened in the developing countries [4]. Moreover, 90% of the neonatal deaths are registered in developing countries too [5]. According to the Ethiopian Demographic and Health Survey (EDHS) 2011, the maternal mortality ratio is 676 per 100,000 live births [6].

Postpartum contraceptive use is defined as the avoidance of closely spaced pregnancies and unintended pregnancy during the first 12 months after delivery [7]. By using a contraceptive, mothers can attain their fertility objective by providing for them to gap their pregnancies and frontier numbers of births [8]. Evidences have shown that encouraging early antenatal care visits, postnatal care, institutional deliveries, and contraceptive adoption are the key elements in improving safe motherhood [1, 9]. In particular, the postpartum period is critical and is the time when many routine interventions are provided to mothers; besides, during the postpartum period, most mothers want to delay or stop the next pregnancy for reducing the risks of short spaced, unwanted pregnancies and associated fetomaternal bad outcomes [10–12].

As the first mainstay of safe motherhood and an essential component of primary health care, contraceptive plays a key role in reducing maternal and newborn morbidity and mortality by preventing unintended pregnancy and short birth intervals [13]. According to the WHO technical consultation committee for better maternal and child health outcomes, an interval of at least 2 years following a live birth is recommended before becoming pregnant again [5]. Pregnancies occurring within a year of the mother's previous birth are riskier for the health of both the mother and the child than those occurring later [14], and children born within one year of a previous birth have a higher risk of mortality than those born after longer intervals [15]. Closely spaced births are also associated with increased chances of chronic undernourishment, stunted growth, and infant mortality [16].

Expanding access to family planning is an effective strategy for saving women's and children's lives and improving their health [17]. Family planning empowers women and households to make decisions when to have children as well as the desired family size. For Africa to tie together the demographic surplus, one of the key initiatives that have been identified as a major policy action is the promotion of modern family planning [17, 18].

Worldwide, more than 90 percent of mothers need to hold up or evade pregnancy within the first year of the postnatal period [19]. Conversely, the majority of women start sex before the first menses next to childbirth devoid of contraceptive use [20–22]. Even if the Sub-Saharan African region had the highest fertility globally, modern contraceptive utilization is very little because communities are still under traditional methods of family planning which lag introduction of modern contraceptives, while contraception is a culturally acceptable norm [20, 23–25]. Actually, postpartum family planning (PPFP) utilization is inconsistent in Sub-Saharan countries, for example, less than 10% in Ethiopia, 15% in Nigeria, 20% in Tanzania, 25% in Kenya, and 40% in Zambia [26].

In Ethiopia, risk of pregnancy among mothers who are sexually active in 12-23 months of the postnatal period is 72%, but it decreases to 64% and 37% for mothers in 6–11 and first 6 months of the postnatal period, respectively [27]. Although the preponderance of postpartum mothers point out the need to utilize contraceptives, contraceptive uptakes are often not obtainable or in use by the first year of the postpartum period. The contraceptive uptake during the postpartum period in Gondar, Ethiopia, was 48.4 [1] which is lower than the 2011 EDHS report for urban women [28].

In Ethiopia, a lot of fragmented studies have been conducted to assess the prevalence and determinants of postpartum contraceptive use. These detached studies reported that prevalence of postpartum contraceptive use in Ethiopian ranged from 10.3% to 80.3% [1, 9, 12, 18, 29–42]. From the reports of these studies, there was a great variation and inconsistency related to the prevalence of postpartum contraceptive use throughout the country [1, 9, 12, 18, 28–42]. In addition to prevalence, sociodemographic (mother educational level), and other determinants like antenatal care (ANC), resumed sexual activities, postnatal care (PNC), menses return, and duration after delivery were the most common determinants reported by the Ethiopian studies [1, 30, 31, 33, 34, 40, 42].

The reasons for the above variation in the prevalence and determinants of postpartum contraceptive use among Ethiopian mothers have not yet been investigated. Therefore, the main aim of this systematic review and meta-analysis was to estimate the pooled prevalence of postpartum contraceptive use and to identify its determinants among postpartum mothers in Ethiopia. The findings of this meta-analysis will help stakeholders and other concerned bodies to plan and fight against unfavorable consequences of short birth interval and unintended pregnancy, furthermore assist to alert the population as a whole, especially the reproductive age group of women. Once more, this meta-analysis will have prime significance for researchers to do interrelated topics. The review question is: What is the top reachable evidence on the prevalence and determinants of postpartum contraceptive use in postpartum mothers in Ethiopia?

## 2. Methods

### 2.1. Identification and Study Selection

A comprehensive and exhaustive search strategy was made by two of the investigators to identify all relevant primary studies. Potentially pertinent studies were identified through a literature search of MEDLINE through PubMed, Science Direct, EMBASE, HINARI, and Cochrane Library. Population, Intervention, Comparison, and Outcome (PICO) format was used to search pertinent studies. The searching was carried out from the 13^th^ of April to the 23^rd^ of May 2019. All papers published in English till May 23^rd^ of 2019 were included in the review. From Google and Google Scholar, unpublished studies have been retrieved. The search was done by the following keywords “Prevalence” OR “Epidemiology” AND “Postpartum contraceptive use” OR “family planning use at postpartum epoch” OR “birth control” AND “Ethiopian”. The search terms were used independently and in amalgamation using Boolean operators like “Or” or “AND.” The systematic review and meta-analysis was carried out in accordance with the Preferred Reporting Items for Systematic Reviews and Meta-Analyses (PRISMA) guideline [43] ([Supplementary-material supplementary-material-1] Table).

## 3. Eligibility Criteria

### 3.1. Inclusion Criteria

#### 3.1.1. Study Area

Only studies conducted in Ethiopia were included.

#### 3.1.2. Publication Condition

Both published and unpublished articles were considered.

#### 3.1.3. Study Design

Epidemiological study design done in Ethiopia including cross-sectional, comparative cross-sectional, case controls, and cohort which contain original data reporting the prevalence and determinants of postpartum contraceptive use among postpartum mothers in Ethiopia was considered.

#### 3.1.4. Language

Both published and unpublished studies in the English language were included.

#### 3.1.5. Population

Articles on postpartum mothers were considered.

### 3.2. Exclusion Criteria

Prior to exclusion, we read the titles and abstracts for the eligibility of the studies. Next to reading the abstracts, if the studies are applicable to our review, we scrutinized the full texts. We excluded articles, which were not fully accessed, after we make contact with the primary author two times through email because we were incapable to examine the quality of each article in the lack of complete text. Furthermore, after deep thorough reviewing of the article, those studies which had not our outcome of interest were excluded.

### 3.3. Data Abstraction

Data were abstracted from incorporated original studies by two members of the study team (TM and BM) separately and all the required information was pulled out using a standardized data abstraction format. The following information was abstracted from each original study: region in which studies were conducted in the country, first author, the district/town where the studies were done, publication year, sample size, study design, response rate, and prevalence of postpartum contraceptive use. For the second objectives (determinants), the information abstraction format was prepared for each specific determinants, i.e., ANC, resumed sexual activity, PNC, mothers' educational level, menses return, and duration after delivery. Variables in this meta-analysis are selected because they are the most repeatedly reported determinants in each study. In this study, we considered variables as a determinant if at least three or more studies reported them as a determinant. Any variance between the two authors during abstraction were argued and resolved through agreement.

### 3.4. Outcome Measurements

The primary objective of this study was the prevalence of postpartum contraceptive use among Ethiopian mothers. The prevalence was calculated by dividing the number of mothers who used postpartum contraceptive to the total number of mothers who have been included in the study (sample size) multiplied by 100. The second objective of the study was to identify the determinants of postpartum contraceptive use. Regarding determinant variables, we calculated the odds ratio from the primary studies using the two by two tables.

### 3.5. Quality Assessment

The scientific strength and quality of each incorporated original study was assessed by the Newcastle-Ottawa Scale tool adapted for cross-sectional study quality assessments [44]. The tool has a pointer that comprises three core parts; the first part of the tool rated as five-star focuses on the methodological quality of each study. The second part of the tool weighed up the equivalency of the primary studies included in meta-analysis. The final part of the tool assessed the quality of primary articles in statistical analysis and outcome point of view. Separately, the two authors weighed up the qualities of the primary studies. The quality of the studies was weighed up by means of these pointers; those with medium (satisfying 50% of quality evaluation criteria) and high quality (≥6 out of 10 scales) were incorporated for analysis. By considering the average score of the two investigators, differences of their evaluation outcomes were resolved.

### 3.6. Statistical Analysis

The individual studies were concisely described via Microsoft Excel format. The summary table specifically illustrated the characteristics of the incorporated primary articles and the core results. We performed the quantitative synthesis via the STATA Version 14.0 (software). Tables as well as forest plots were used to present the meta-analysis results. Primary article standard error of prevalence was computed by using the binomial distribution formula. Statistical heterogeneity was tested via the Chi^2^ test (Cochran *Q* test) at a *p* value ≤ 0.05. The heterogeneity level was quantified with the *I*^2^ statistics [45] (*I*^2^ = 99.4%, *p* < 0.001); therefore, considerable heterogeneity was assumed, and Mantel-Haenszel random effects model meta-analysis was employed to estimate the DerSimonian and Laird's pooled effect. Publication bias was also examined by performing Egger's correlation and Begg's regression intercept tests at a 5% significant level [46, 47]. The results of these tests indicated that there was no publication bias as evidenced by *p* = 0.257 in Egger's test. Furthermore, to reduce the random variations among the point estimates of the primary study, subgroup analysis was done based on the study area (residence) and region.

## 4. Results

### 4.1. Search Results

**Figure 1:**
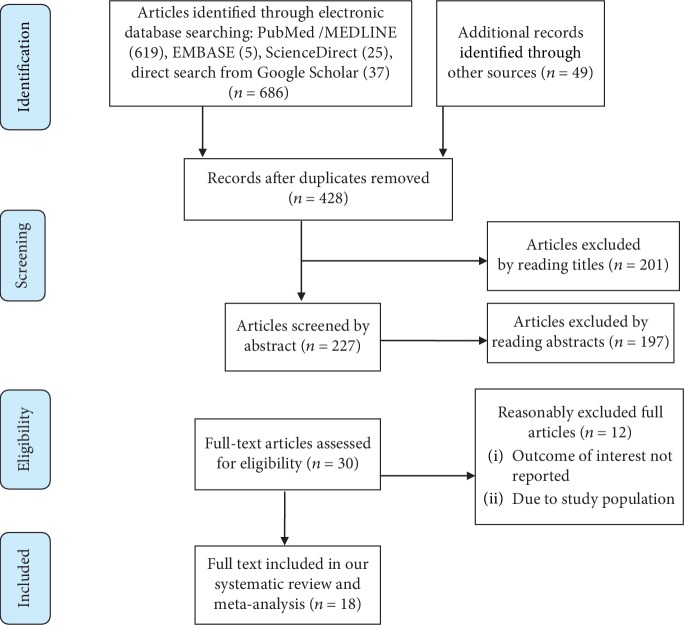
Flow chart describing the selection of studies for the systematic review and meta-analysis of prevalence and determinants of postpartum contraceptive use among mothers in Ethiopia, 2019.

From all source of the databases, a searching total of 735 primary articles were accessed concerning prevalence and determinants of postpartum contraceptive use among mothers. 307 of these were duplicates consequently excluded. After reviewing the title and abstract, 398 articles were excluded as inappropriate to the current systematic review and meta-analysis. Thus, there were 30 full-text articles appropriately eligible according to preset criteria. Additionally, 12 primary articles were excluded because of the study population and outcome of interest. Among these, eight of the studies were conducted in Uganda [48, 49], Ghana [50, 51], Burundi and Rwanda [52], Thailand [53], India [54], and Kenya [55]. From the remaining four studies, one was conducted in Addis Ababa, Ethiopia [56] and excluded because of the unreported outcome of interest. However, the other three were conducted at the entire Ethiopia and excluded because the studies were not specific which are not parallel to the article that we use in our analysis [28, 57, 58]. Lastly, 18 studies were alleged to be eligible and included in the meta-analysis (Figure 1).

#### 4.1.1. Original Article Characteristics

The 18 original articles included in the present systematic review and meta-analysis are characterized and summarized in Table 1. All of the articles which are part of the present systematic review and meta-analysis are cross-sectional in study design. The sample size ranged from 248 in Debre Berhan town [27] to 899 in Dabat district [33]. Regarding the study period, all the primary studies were conducted from 2015 to 2019. In the present meta-analysis, to estimate the pooled prevalence of postpartum contraceptive use entirety, 10,630 postpartum mothers were drawn in. Regarding geographical distribution of the studies, the 18 studies were found from the six regions of the country: six from Amhara [1, 12, 33, 35, 36, 41], four from Tigray [29, 31, 32], three studies from Addis Ababa [30, 38, 40], three from Southern Nations, Nationalities, and Peoples' Region (SNNPR) [9, 18, 42], one from Oromia region [34], and one from Somali region [37]. On the other hand, there were no studies conducted in Gambella, Harari, Afar, and Benishagul Gumuz regions of the country. The highest prevalence of postpartum contraceptive use (80.3%) was reported in Addis Ababa [30] while the lowest prevalence (10.3%) was reported from a study done in Dabat district (Amhara region) [33]. In relation to the response rate, the primary articles incorporated in the meta-analysis range from 94.9% to 100%, and essentially, all the studies had a good response rate. Three [18, 38, 40] of the 18 studies were unpublished while the other 15 studies were published in reputable journals. Lastly, the primary studies included in the present systematic review and meta-analysis had a quality score of 6–9 out of 10 points.

### 4.2. Meta-analysis

**Figure 2:**
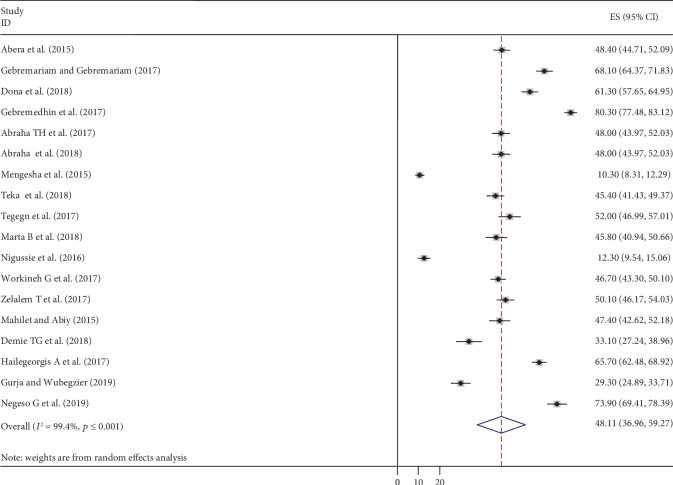
Forest plot of the pooled prevalence of postpartum contraceptive use in Ethiopia, 2019.

The pooled prevalence of postpartum contraceptive use among mothers in the postpartum period in Ethiopia was 48.11% (95% CI: 36.96, 59.27) as shown in the forest plot (Figure 2). There was extreme heterogeneity showed across the studies and exposed by *I*^2^ statistic (*I*^2^ = 99.4, *p* value < 0.001). Hence, to estimate the pooled prevalence of postpartum contraceptive use among mothers in Ethiopia, random effects model meta-analysis was used.

Moreover, to identify the possible sources of heterogeneity, we executed a univariate metaregression model by allowing different factors associated with the heterogeneity, such as publication year and sample size; however, none of these variables was established to be statistically significant. Begg's and Egger's tests confirmed the absence of statistically significant publication bias (*p* = 0.472 and *p* = 0.057, respectively).

### 4.3. Subgroup Analysis

**Figure 3:**
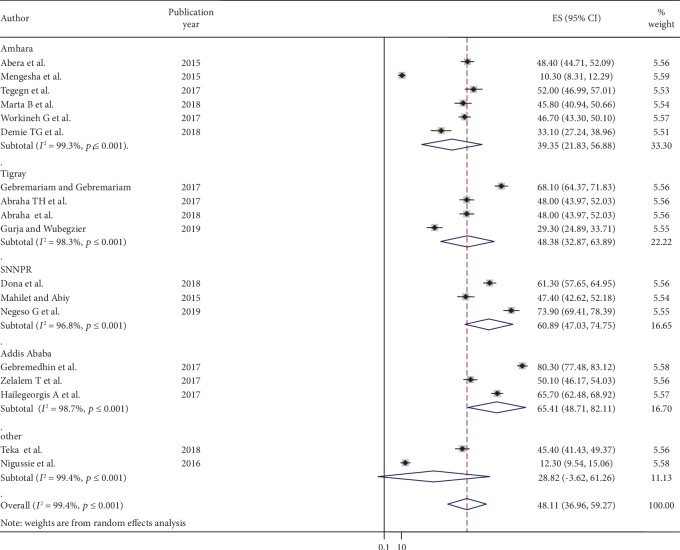
The subgroup analysis of postpartum contraceptive use status by regional states in Ethiopia, 2019.

Subgroup analysis was executed based on the regions where the studies were conducted (administrative state within the country) in our current meta-analysis. Accordingly, the highest prevalence was observed in Addis Ababa with a prevalence of 65.41% (95% CI: 48.71, 82.11) followed by SNNRP region, 60.89% (95% CI: 47.03, 74.75) and Tigray 48.38% (95% CI: 32.87, 63.89) (Figure 3).

**Figure 4:**
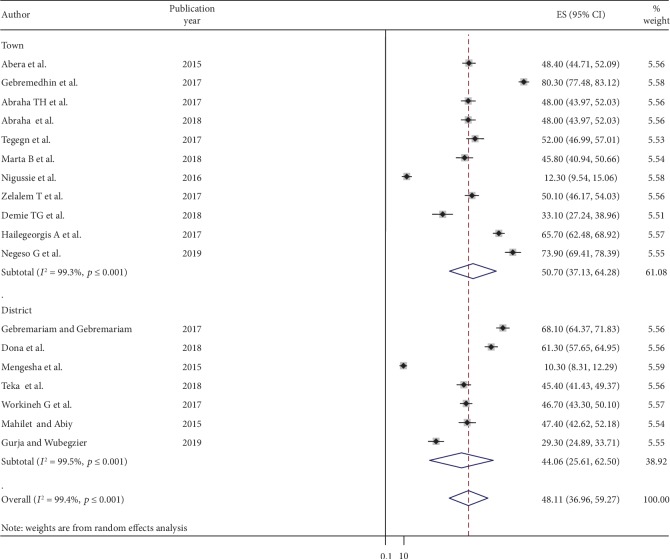
The subgroup analysis of postpartum contraceptive use status by study settings (town and district) in Ethiopia, 2019.

Besides, we also executed subgroup analysis based on the residential area where (town and district) the primary studies were conducted. From the result of this subgroup analysis, we found that studies conducted in town, 50.7% (95% CI: 37.13, 64.28) were slightly higher in family planning use as compared to those studies conducted at the district area, 48.11% (95% CI: 36.96, 59.27) (Figure 4).

### 4.4. Determinants of Postpartum Contraceptive Use

**Figure 5:**
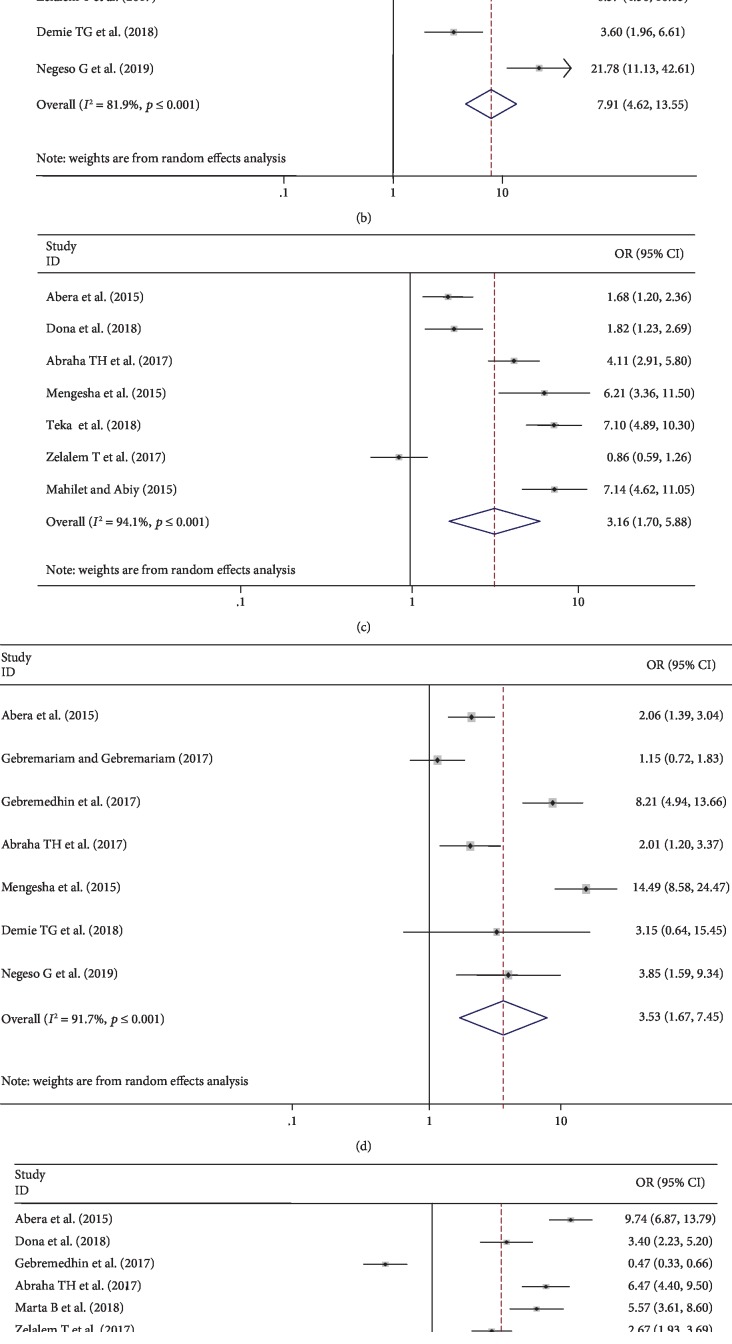
Forest plot depicting pooled odds ratio (log scale) of the associations between postpartum contraceptive use and its alleged determinants: (a) ANC, (b) resumed sexual activity, (c) PNC, (d) mothers' educational level, (e) menses resumption, and (f) duration after delivery.

Using a total of thirteen primary studies [1, 9, 12, 18, 29–31, 33, 34, 36, 37, 40, 42] with data that can be analyzed, we have examined the associations of determinants (ANC, resumed sexual activities, PNC, mother educational level, menses return, and duration after delivery) with our outcome variable (postpartum contraceptive use) (Figure 5). None of the studies has shown significant differences in all the executed determinant sensitivity analysis. Hence, from the ten studies, we found that ANC utilization showed a significant association with postpartum family planning use.

In epidemiological explanation, this showed us that mothers who had ANC follow-up were 4.89 times more likely to use postpartum family planning as compared to those mothers who had no ANC follow-up during pregnancy with odds ratio 4.89 (95% CI: 2.34–10.21) (Figure 5(a)).

Six studies also indicated that resumed sexual intercourse was strongly associated with the postpartum family planning use (Figure 5(b)). Those mothers who had started sexual intercourse after delivery were 7.91 times more likely to use postpartum contraceptive as compared to their counterparts with odds ratio of 7.91 (95% CI: 4.62, 13.55).

Furthermore, results from the meta-analyses of the studies (Figure 5(c)) have also revealed that the PNC follow-up was a significant factor associated with postpartum contraceptive use of the mothers. Mothers who had a PNC follow-up were 3.16 times more likely to use family planning as compared with those mothers who had no PNC follow-up with odds ratio 3.16 (95% CI: 1.7, 5.88).

Seven studies also indicated that secondary and above level of education as well as postpartum period ≥ 6 months after childbirth were strongly associated with the family planning use during the postpartum epoch (Figures 5(d) and 5(f)), respectively. Those mothers who had secondary and above level of education as well as postpartum period ≥ 6 months after childbirth were 3.53 and 5.59 times more likely to use family planning during postpartum compared to their counterparts, respectively; for secondary and above level of maternal education, odds ratio is 3.53 (95% CI: 1.67, 7.45), and for ≥6 months after childbirth, odds ratio is 3.59 (95% CI: 2.39, 5.4).

Menses resumption was another significant factor associated with postpartum contraceptive use (Figure 5(e)). Mothers who had menses were 3.12 times more likely to use postpartum family planning as compared to mothers who had no menses, odds ratio 3.12 (95% CI: 1.52, 6.39).

## 5. Discussion

The pooled prevalence of postpartum contraceptive use among postpartum mothers in the current meta-analysis study indicated that almost near one in two 48.11 (95% CI: 36.96, 59.27) mothers have been adopted in the utilization of modern contraceptive.

It is obvious that postpartum contraceptive use provides numerous socioeconomic and healthy outcomes within the society as well as the country at large by decreased maternal mortality, miscarriage or abortion, and newborn and child morbidity and mortality since it has been associated with unintended pregnancy and closely spaced birth [7, 59, 60]. WHO describes the postpartum epoch as the most critical and yet the most neglected phase in the lives of mothers and babies; even most deaths occur during the postpartum epoch [2]. Despite the fact that uptake of PPFP remains low in Sub-Saharan Africa and very little is known about how pregnant women arrive at their decisions to adopt PPFP, benefit of early adoption or continuation of family planning is well known and is positive [48, 61, 62]. Thus, it is a dual load for those countries still below the poverty level as allocating resources for the citizen becomes a headache for the government.

Even if there was no analogous meta-analysis study conducted on this specific research question within the area, the prevalence reported in the present study is incomparable with the survey carried out at the time of 6 months after birth, in Malawi is 86% [63] and in America is 88% [64]. However, the prevalence of postpartum contraceptive use is more than that reported for Uganda (28%) [48] and Burundi (20%) [52]. The potential explanation could be attributed to the difference in socioeconomic status, sociocultural values, norms, religious beliefs, and study setting and area. Similarly, our result is substantially lower as compared with the review conducted at Chiang Mai University Hospital, Thailand (97.6%) [53], and Rwanda (51.1%) [52]. This divergence might be due to the differences in health policy of the respective countries, difference in culture and study design, accessibility of contraceptives, male involvement, and study participant level of understanding towards the health benefits of using a postpartum contraceptive.

The subgroup analysis of this study showed significant variation among Ethiopian regional states with respect to postpartum contraceptive use. As the result, the highest prevalence of postpartum contraceptive use was observed in Addis Ababa, 66.41% (95% CI: 48.71, 82.11), followed by SNNPS region, 50.89% (95% CI: 47.03, 74.75), while the lowest prevalence was noticed in other regions in this case with a prevalence of 28.82% (95% CI: -3.62, 61.26). The potential rationalization for this variation might be associated with cultural, religious, social, and economical differences across the regions. The other potential rationalization for the higher prevalence of postpartum contraceptive use in Addis Ababa could be due to the differences in the study settings, such as better health facility and access to family planning education through media, for example, in Addis Ababa City, there are a lot of posters, banners, television, and radio programs about the importance of postpartum contraceptive use, thus internalized by the society.

Concerning the study setting (residence), the prevalence of postpartum contraceptive use was slightly higher in those studies conducted in the urban (town) area. The possible explanations for this variation might be due to sociocultural and socioeconomic differences between the town and districts. The other possible explanation for higher utilization of PPFP in towns might be due to differences in the study settings, such as better health facility and access, easy access to digital as well as nondigital media in town for them to be familiarized with the advantage of using family planning during the postpartum epoch and its associated health outcomes both for the mother and her child as compared to district areas. The other very important factor for this discrepancy may be due to active and sympathetic partner involvement in urban areas compared with rural areas.

The present study was also aimed at identifying the determinants of postpartum contraceptive use among Ethiopian mothers based on the reports of primary studies. In the current meta-analysis, secondary and above level of maternal education, ANC, resumed sexual activities, PNC, menses return, and 6-9 months of duration after childbirth were found to be determinants of use of postpartum contraceptive. Accordingly, in this study, postpartum contraceptive use was significantly higher in secondary and above level of educated mothers as compared to those mothers who had no formal education.

This finding is consistent with studies conducted in Rwanda [52], Uganda [48], and Burundi [52]. This significant difference between mothers who had no formal education as compared to secondary and above level of education could be explained by the fact that educated mothers have better understanding of benefits of contraception, risks of short interval pregnancies, and socioeconomical burdens.

Moreover, those mothers who were educated know the range of benefits of contraceptive other than their primary purpose of pregnancy prevention like reducing pregnancy-related maternal and neonatal morbidity and mortality and trimming down the risk of developing certain reproductive cancers and its usage to treat many menstrual-related symptoms and disorders. Therefore, we have to strive and promote women's education in Ethiopia to excel PPFP use and diminish maternal and neonatal morbidity/mortality.

Menses resumption after childbirth was also another determinant for PPFP use. Mothers whose menses resumed after childbirth were more predisposed to use family planning as compared to those mothers whose menses did not resume. This finding is in line with different studies carried out on family planning [51]. This might be explained by the perception of the mothers that once menses have returned, the risk of pregnancy is increased, and this prompts them to use contraceptive methods whereas ammenhorric women would underestimate the risk of pregnancy by assuming that amenorrhea could guarantee protection against pregnancy regardless of the time of the postpartum period. Additionally, majority of the mothers in Ethiopia have no access to gain knowledge about human reproductive physiology; also, they do not know when they are potentially able to get pregnant and use family planning. As the result, menses resumption is considered as the critical time to take contraceptive during the postpartum epoch.

Similarly, those mothers whose postpartum epoch was six months and above after childbirth were more likely to use family planning as compared to those mothers whose postpartum epoch was less than six months after childbirth. This finding is supported by previous studies conducted in Kenya [55] and Ethiopia [28]. This might be due to the fact that as the duration of the postpartum period increases, mothers engage in sexual activity, and moreover, their menses will resume. Hence, women might fear getting pregnant which boosts them to use a contraceptive. In addition, resumed sexual activity was the other variable that showed statistically significant association with PPFP use. This finding is in line with different studies carried out on family planning [51]. This might be due to the fact that women who resumed sexual intercourse have a fear of getting pregnant. Therefore, they inquire contraception compared with those who had not resumed sex. Lastly, in this meta-analysis, we observed that those mothers who had ANC and PNC follow-up were more likely to use contraceptive as compared to their counterparts, respectively. This finding is supported by previous studies conducted in Kenya [55], Uganda [48], Rwanda [52], and America [64]. These studies reported that having antenatal as well as postnatal care services were positively associated with contraceptive uses. Another explanation for this finding could be justified by the fact that women might get the opportunity for contraceptive counseling and more other associated information from health professionals during ANC and PNC follow-up so as to use family planning in an effective and timely manner.

### 5.1. Limitations of the Study

The study designs for all primary articles incorporated in this review were cross-sectional in nature; as the result, the confounding variables most of the time might affect the outcome variable. Concerning sample size, some of the studies included in this review had a not as such large sample size and may influence the estimated report. Another assuming factor that could limit this nation-based review was the fact that it considered only articles published in the English language. Moreover, this meta-analysis symbolized only studies reported from seven regional states of the country. Hence, the people in those regions which were not included in the review may be unrepresented and affect the pooled prevalence of postpartum contraceptive use.

## 6. Conclusions

The study found that the prevalence of postpartum contraceptive use among mothers still did not reach the desired level, with slightly lower than 1 in 2 mothers utilizing family planning. Secondary and above level educated mother, menses resumption, six to nine months after delivery, resumed sexual intercourse, and having ANC and PNC were found to be determinants of PPFP. Hence, based on our findings, we powerfully advised that a special contemplation shall be given to mothers. In addition, the health sector policy maker, promoters, and providers as well as other concerned stakeholders should launch family planning education programs about the health benefits of using postpartum contraceptives especially to prevent unintended pregnancy and extend interpregnancy interval.

## Figures and Tables

**Table 1:** Descriptive summary of 18 primary studies showing the prevalence and determinants for postpartum contraceptive use among postpartum mothers in Ethiopia included in the systematic review and meta-analysis, 2019.

Region	Town/district	Author	Publication year	Sample size	Response rate (%)	Quality score (10 pt)	Prevalence (95% CI)
Amhara	Gondar	Abera et al. [1]	2015	705	99.7	8	48.4 (44.71, 52.10)
	Dabat	Mengesha et al. [33]	2015	899	100	9	10.3 (8.31, 12.29)
	Dessie	Tegegn et al. [35]	2017	383	99.7	7	52 (46.99, 57.01)
	Gondar	Berta et al. [36]	2018	404	100	8	45.8 (40.94, 50.66)
	Gozamen	Workineh et al. [41]	2017	844	98.22	6	46.7 (43.30, 50.10)
	Debre Berhan	Demie et al. [12]	2018	248	100	7	33.1 (27.24, 38.96)

Tigray	Ganta-Afeshum	Gebremariam and Gebremariam [29]	2017	605	95.5	8	68.1 (64.37, 71.83)
	Axum	Abraha et al. [31]	2017	601	98.2	8	48 (43.97, 52.03)
	Axum	Abraha et al. [32]	2018	604	97.7	7	48 (43.97, 52.03)
	Tahtay Koraro	Embafrash and Mekonnen [39]	2019	422	96.9	7	29.3 (24.89, 33.71)

Addis Ababa	Addis Ababa	Gebremedhin et al. [30]	2017	803	94.9	8	80.3 (77.48, 83.12)
	Addis Ababa	Tedla et al. [40]	2017	631	98.7	7	50.1 (46.17, 54.03)
	Addis Ababa	Ross and Winfrey et al. [19]	2017	845	98.6	8	

SNNPR	Aroressa	Gebremariam and Gebremariam [29]	2018	695	98.4	8	61.3 (57.65, 64.95)
	Hossana	Gejo et al. [42]	2019	368	100	7	73.9 (69.41, 78.39)
	Butajjira	Abiy [18]	2015	421	99.7	6	47.4 (42.62, 52.18)

Oromia	Gida Ayana	Teka et al. [34]	2018	616	97.8	8	45.4 (41.43, 49.37)
Somali	Kebribeyah	Nigussie et al. [37]	2016	556	98	6	12.3 (9.54, 15.06)

## Supplementary Materials

Supplementary Materials PRISMA (Preferred Reporting Items for Systematic Review and Meta-Analysis) 2015 checklist: recommended items to address in a systematic review protocol^∗^.
